# Complete chloroplast genome of *Sanguisorba* × *tenuifolia* Fisch. ex Link

**DOI:** 10.1080/23802359.2018.1501326

**Published:** 2018-08-17

**Authors:** Inkyu Park, Sungyu Yang, Wook Jin Kim, Pureum Noh, Hyun Oh Lee, Byeong Cheol Moon

**Affiliations:** a Herbal Medicine Research Division, Korea Institute of Oriental Medicine, Daejeon, Republic of Korea;; b Phyzen Genomics Institute, Seongnam, Republic of Korea

**Keywords:** *Sanguisorba × tenuifolia* Fisch. ex Link, medicinal plant, chloroplast genome, Rosaceae

## Abstract

*Sanguisorba* × *tenuifolia* Fisch. ex Link is an important herbal medicine. To facilitate species identification, we sequenced its complete chloroplast genome using the Illumina MiSeq platform. The data show that the chloroplast genome of *S*. × *tenuifolia* is 155,403 bp in size, comprising an 85,525 bp large single-copy (LSC) region, a 18,726 bp small single-copy (SSC) region, and two inverted repeats (IR) regions, IRa and IRb (each 25,576 bp). The genome contains 112 unique genes, including 79 protein-coding genes, four ribosomal RNAs genes, and 30 transfer RNAs genes. Phylogenetic analysis revealed that *S*. × *tenuifolia* is most closely related to *Hagenia abyssinica*.

*Sanguisorba* × *tenuifolia* Fisch. ex Link, a member of the Rosaceae family, is widely distributed in China and Korea. Dried roots of *S.* × *tenuifolia*, called Sanguisorbae Radix (SR), are used as a Korean traditional herbal medicine to treat diarrhoea and bleeding (KIOM 2018). Recent pharmacological reports of SR in alloxan-induced diabetic rats indicate hypoglycaemic effects (Kuang et al. 2011). Moreover, SR are frequently mixed and indiscriminately misused with other similar plant species in Korean herbal markets. Thus, to accurately identify *S.* × *tenuifolia* and discriminate it from other similar species, we sequenced its complete chloroplast genome.

Fresh leaves of *S.* × *tenuifolia* were collected from its native habitat in Korea (34°33*'*39.7*"*N and 126°33*'*43.5*"*E). Specimens were labelled with unique identification numbers and registered in the Korean Herbarium of Standard Herbal Resources (Index herbarium code KIOM) at the Korea Institute of Oriental Medicine (KIOM), with voucher number KIOM201201005142. Genomic DNA was extracted from leaf samples using a DNeasy Plant Maxi Kit (QIAGEN, Valencia, CA). An Illumina paired-end library was constructed and sequenced using the MiSeq platform (Illumina Inc., San Diego, CA). The complete chloroplast genome of *S.* × *tenuifolia* was deposited in the GenBank database of the National Center for Biotechnology Information (NCBI) under accession number MH513641.

Illumina sequencing of *S.* × *tenuifolia* yielded 1.4 Gb of high-quality paired-end reads. The chloroplast genome sequence contigs of *S.* × *tenuifolia* were assembled *de novo* from low-coverage whole-genome sequences (Kim et al. 2015). The complete chloroplast genome of *S.* × *tenuifolia* comprised 161,629 bp. The chloroplast genome showed a typical quadripartite structure comprising a large single-copy (LSC) region of 85,525 bp, a small single-copy (SSC) region of 18,726 bp, and two inverted repeat (IR) regions, IRa and IRb (each 25,576 bp). The GC content of the chloroplast genome was 37.2%, with the IR regions showing a higher GC content (42.7%) than the LSC (35.2%) and SSC (31.3%) regions. These data indicate that the chloroplast genome of *S.* × *tenuifolia* is AT-rich, which is consistent with the chloroplast genomes of other plant species (Park et al. 2017). The chloroplast genome of *S.* × *tenuifolia* harboured 112 unique genes, including 79 protein-coding genes, 30 genes encoding transfer RNA (tRNAs), and four genes encoding ribosomal RNAs (rRNAs). Of the 112 genes, 17 were duplicated in the IR regions and 18 contained introns. Among the genes containing introns, 16 contained a single intron and two (*ycf3* and *clpP*) harboured two introns.

To investigate the phylogenetic relationship between *S.* × *tenuifolia* and other plant species, we aligned the nucleotide sequences of 70 protein-coding genes of *S.* × *tenuifolia* with those of homologs from ten other taxa, spanning a total length of 65,361 bp. The phylogenetic tree constructed using the maximum likelihood (ML) method contained nine nodes, each with bootstrap values of 100% (Figure 1), indicating a strong phylogenetic relationship with the Rosaceae family. Additionally, the phylogenetic tree revealed that *S.* × *tenuifolia* formed a monophyletic group with *Hagenia abyssinica* within Rosaceae, with bootstrap support values of 100% (Figure 1).

**Figure 1.**
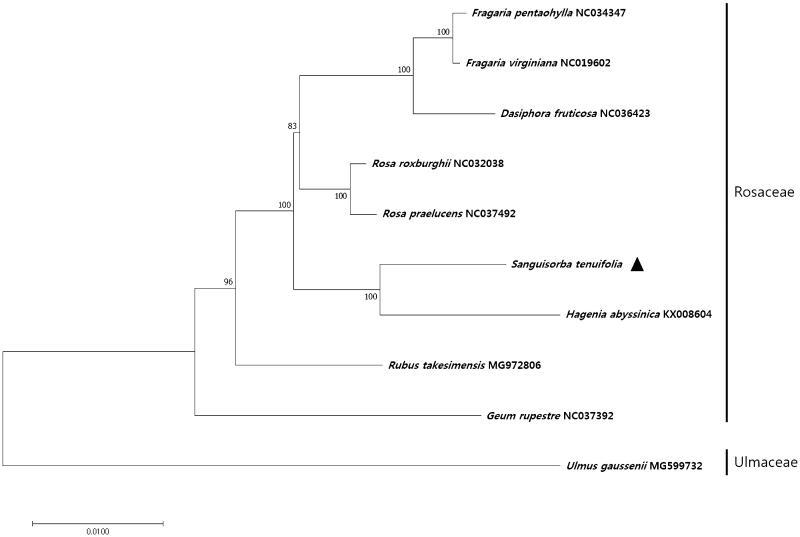
Maximum likelihood (ML) tree based on the chloroplast protein-coding genes of 10 taxa, including *S.* × *tenuifolia* and one outgroup taxon. Seventy protein-coding genes were aligned using MAFFT (Katoh et al. 2002). ML analysis was performed using MEGA6, with 1000 bootstrap replicates (Tamura et al. 2013). The bootstrap support values from 1000 replicates are indicated at the nodes.
